# Cell cycle perturbations and apoptosis induced by isohomohalichondrin B (IHB), a natural marine compound

**DOI:** 10.1038/sj.bjc.6690044

**Published:** 1999-01

**Authors:** D Bergamaschi, S Ronzoni, S Taverna, M Faretta, P De Feudis, G Faircloth, J Jimeno, E Erba, M D'Incalci

**Affiliations:** Department of Oncology, Cancer Pharmacology Laboratory, Istituto di Ricerche Farmacologiche ‘Mario Negri’, via Eritrea 62, Milan, 20157, Italy; PharmaMar USA Inc., 26 Landsdowne Street, Cambridge, MA, USA; PharmaMar SA, Research and Development, c/Caldera, 28760 Tres Cantos, Madrid, Spain

**Keywords:** natural compound, isohomohalichondrin B, mitotic block, apoptosis

## Abstract

Isohomohalichondrin B (IHB), a novel marine compound with anti-tumoral activity, extracted from the *Lissodendorix* sponge, inhibits GTP binding to tubulin, preventing microtubule assembly. Cell cycle perturbations and apoptosis induced by IHB were investigated on selected human cancer cell lines by using flow cytometric and biochemical techniques. Monoparameter flow cytometric analysis showed that 1 h IHB exposure caused a delayed progression through S-phase, a dramatic block in G_2_M phase of the cell cycle and the appearance of tetraploid cell population in LoVo, LoVo/DX, MOLT-4 and K562 cells. At 24 h after IHB exposure, the majority of cells blocked in G_2_M were in prophase as assessed by morphological analysis and by the fact that they expressed high levels of cyclin A/*cdc2* and cyclin B1/*cdc2*. At 48 h, all cells were tetraploid as assessed by biparameter cyclin A/DNA and cyclin B1/DNA content analysis. Apoptotic death was detected in both leukaemic MOLT-4 and K562 cells, which express wild-type and mutated p53 respectively, when the cells were blocked in mitotic prophase. In conclusion, IHB is a novel potent anti-tumour drug that causes delayed S-phase progression, mitotic block, tetraploidy and apoptosis in cancer cell lines. © 1999 Cancer Research Campaign


*British Journal of Cancer* (1999) **79**(2), 267–277
© 1999 Cancer Research Campaign

### Cell cycle perturbations and apoptosis induced by isohomohalichondrin B (IHB), a natural marine compound

**D Bergamaschi[1], S Ronzoni[1], S Taverna[1], M Faretta[1], P De Feudis[1], G Faircloth[2], J Jimeno[3], E Erba[1] and M D'Incalci[1]**

[1]Cancer Pharmacology Laboratory, Department of Oncology, Istituto di Ricerche Farmacologiche 'Mario Negri', via Eritrea 62 – 20157 Milan, Italy; [2]PharmaMar USA, Inc., 26 Landsdowne Street, Cambridge MA, USA; [3]PharmaMar SA, Research and Development, c/Caldera, 3, 28760 Tres Cantos, Madrid, Spain

**Summary** Isohomohalichondrin B (IHB), a novel marine compound with anti-tumoral activity, extracted from the *Lissodendorix* sponge, inhibits GTP binding to tubulin, preventing microtubule assembly. Cell cycle perturbations and apoptosis induced by IHB were investigated on selected human cancer cell lines by using flow cytometric and biochemical techniques. Monoparameter flow cytometric analysis showed that 1 h IHB exposure caused a delayed progression through S-phase, a dramatic block in $G_2M$ phase of the cell cycle and the appearance of tetraploid cell population in LoVo, LoVo/DX, MOLT-4 and K562 cells. At 24 h after IHB exposure, the majority of cells blocked in $G_2M$ were in prophase as assessed by morphological analysis and by the fact that they expressed high levels of cyclin A/*cdc2* and cyclin B1/*cdc2*. At 48 h, all cells were tetraploid as assessed by biparameter cyclin A/DNA and cyclin B1/DNA content analysis. Apoptotic death was detected in both leukaemic MOLT-4 and K562 cells, which express wild-type and mutated p53 respectively, when the cells were blocked in mitotic prophase. In conclusion, IHB is a novel potent anti-tumour drug that causes delayed S-phase progression, mitotic block, tetraploidy and apoptosis in cancer cell lines.

**Keywords:** natural compound; isohomohalichondrin B; mitotic block; apoptosis

Isohomohalichondrin B (IHB) is a polyether macrolide (Figure 1) isolated from the *Lissodendoryx* sponge and shows in vitro cytotoxic activity against a variety of human cancer cell lines (Hirata and Uemura, 1986) and, more recently, in vivo anti-tumour activity against murine tumours and human tumours transplanted in nude mice (unpublished data). The drug was initially tested in the NCI tumour cell lines screening panel and was found to be effective in inhibiting cell growth at concentrations ranging from $10^{-9}$ to $10^{-5}$ M (unpublished data made available by the NCI). The NCI comparative analysis showed a significant correlation between the pattern of sensitivity of IHB to that of other anti-tubulin drugs such as the vinca alkaloids vincristine and vinblastine and maytansine.

These data are in good agreement with biochemical studies indicating that IHB binds to tubulin, inhibiting GTP binding and preventing microtubule assembly (García-Rocha et al, 1996).

As IHB is a strong potential candidate for clinical investigation, it is of interest to characterize further its mode of action and its pharmacological properties.

In the present study, the in vitro effect of IHB on the cell cycle and the mechanism of cell death have been investigated using selected human cancer cell lines.

### MATERIALS AND METHODS

### Cells and culture conditions

Human leukaemic K562 and MOLT-4 cell lines were grown in suspension in RPMI-1640 medium (Gibco Europe, Paisley, UK) containing 10% heat-inactivated fetal bovine serum (FBS) (Mascia Brunelli SPA, Milan, Italy) in T-25-cm² tissue culture flasks (Costar–Corning–Nucleopore, Cambridge, MA, USA). Human colon LoVo and LoVo/DX were grown in monolayer culture in F-12 (Ham) medium supplemented with 15% FBS and 1% vitamins in T-25-cm² tissue culture flasks in standard culture conditions.

### Drug treatment

IHB was provided by PharmaMar. Exponentially growing LoVo, LoVo/DX, MOLT-4 and K562 cells were treated for 1 h with different concentrations of IHB. The shorter exposure time was chosen in order to evaluate more precisely the time course of the cell cycle perturbations induced by the drug. After treatment, the medium was removed and the cells were washed with phosphate-buffered saline (PBS) and provided with fresh medium. At different times after drug washout, the cells were counted with a Coulter counter (model ZM; Luton, UK), fixed in 70% ethanol and kept at 4°C before analysis by flow cytometry. For 5-bromo-2′-deoxyuridine (BrdU)/DNA biparametric analysis during the last

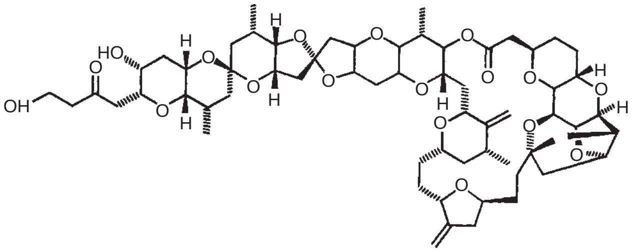

**Figure 1** IHB chemical structure

*Received 20 October 1997*
*Revised 25 April 1998*
*Accepted 30 April 1998*

*Correspondence to:* E Erba

15 min of treatment, the cells were treated with 30 μM BrdU. After treatment, the medium was removed, the cells washed with PBS and provided with fresh medium. At different times after drug washout the cells were counted and fixed as described previously (Erba et al, 1995).

### DNA staining

The fixed cells were washed with cold PBS and stained with 2 ml of propidium iodide (PI) solution containing 25 μg ml$^{-1}$ PI in PBS plus 25 μl of RNAase (Calbiochem, La Lolla, CA, USA), 1 mg ml$^{-1}$ in water, for 60–120 min at room temperature in the dark.

### BrdU/DNA staining

For detection of BrdU incorporation into DNA, the fixed cells were washed with cold PBS and the DNA was denatured with 1 ml of 3 M hydrogen chloride for 20 min at room temperature to allow the anti-BrdU Mab to react with the BrdU incorporated in the DNA chain. DNA denaturation was stopped by adding 3 ml of 0.1 M sodium tetraborate pH 8.5. After centrifugation, the pellet was incubated with 1 ml of 0.5% Tween-20 (Sigma Chemical, St Louis, MO, USA) in PBS and 1% normal goat serum (NGS) (Dakopatts, Denmark) for 15 min at room temperature. The incorporated BrdU was visualized by incubating the cells with the anti-BrdU Mab (Becton Dickinson, Sunnyvale, CA, USA) diluted 1:10 in 0.5% Tween-20 in PBS for 1 h

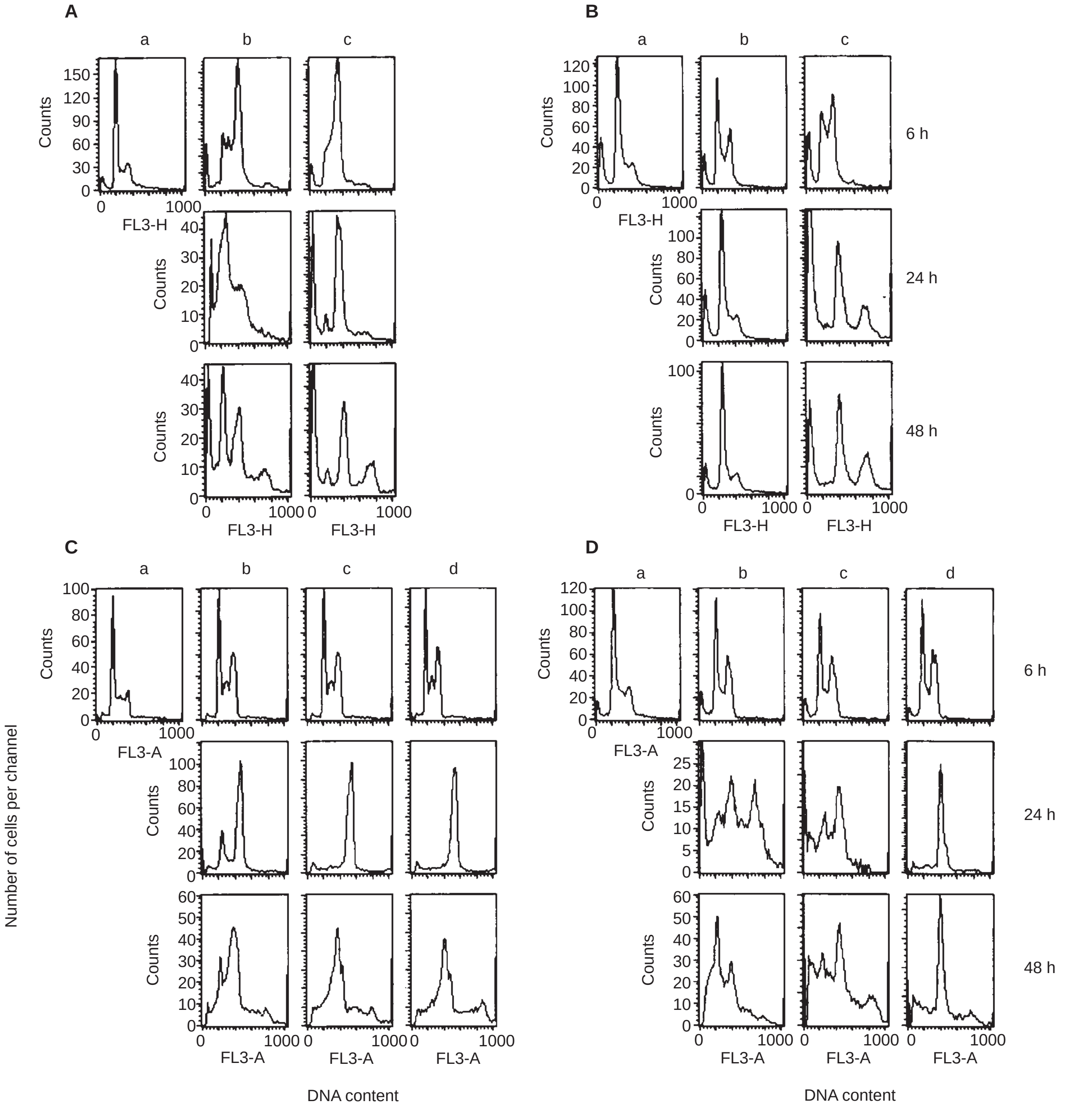

**Figure 2** Effects of IHB on the cell cycle phase distribution evaluated at 6, 24 and 48 h after drug washout. (**A**) LoVo cells: a, control; b, IHB 1 nM; c, IHB 2 nM. (**B**) LoVo/DX cells: a, control; b, IHB 2.5 nM; c, IHB 10 nM. (**C**) MOLT-4 cells: a, control; b, IHB 0.5 nM; c, IHB 1 nM; d = IHB 2 nM. (**D**) K562 cells: a, control; b, IHB 0.5 nM; c, IHB 1 nM; d = IHB 2 nM

*© Cancer Research Campaign 1999*

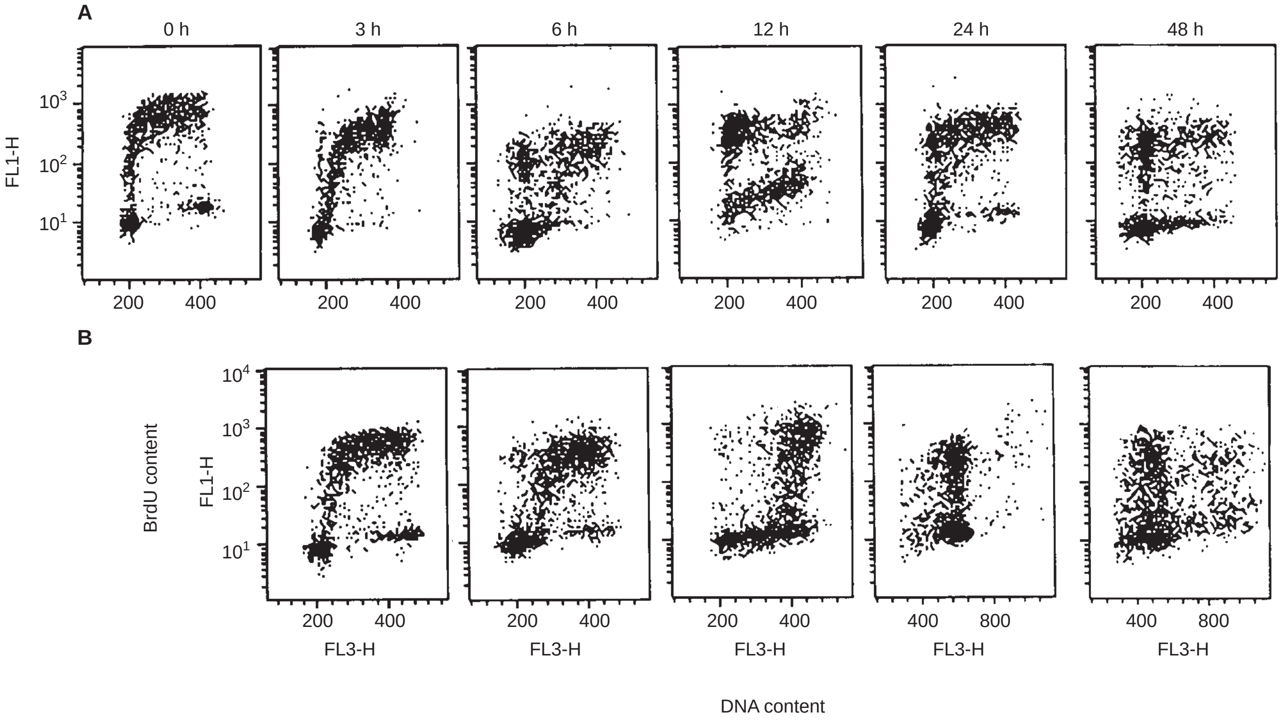

**Figure 3** Biparametric BrdU/DNA flow cytometric analysis of (**A**) MOLT-4 control and (**B**) IHB-treated cells evaluated at different time intervals after drug washout

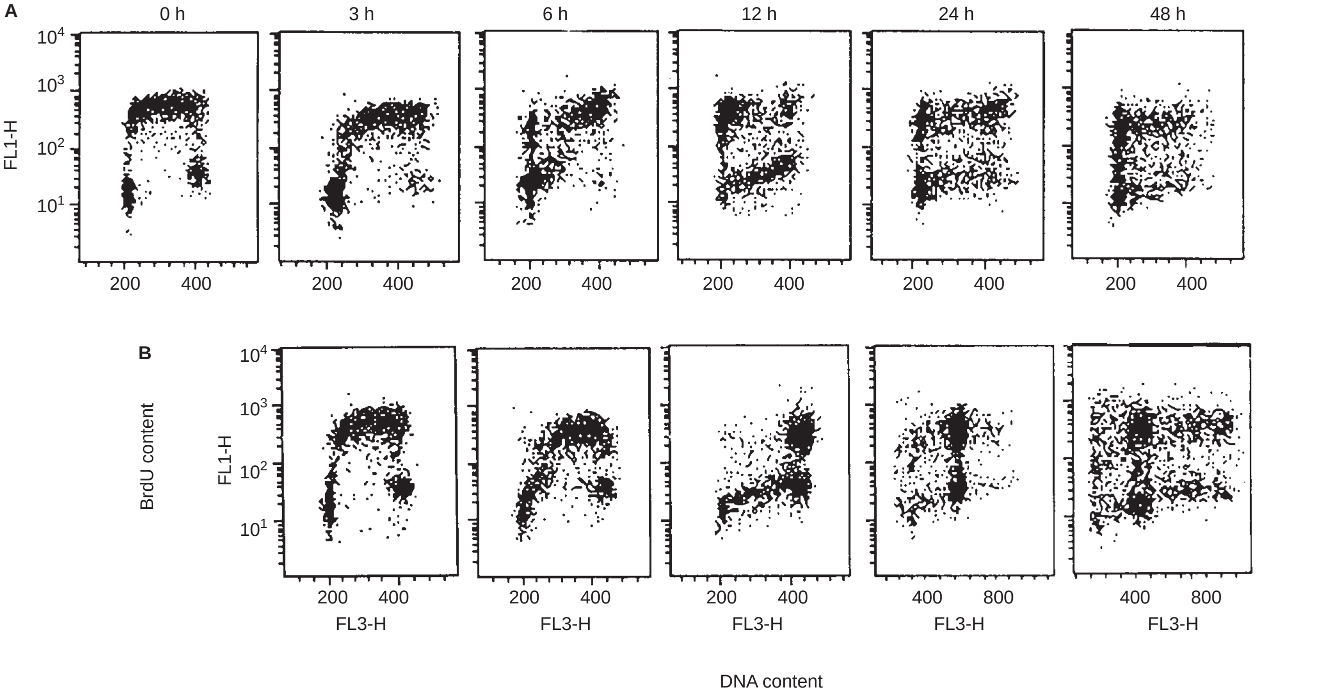

**Figure 4** Biparametric BrdU/DNA flow cytometric analysis of (**A**) K562 control and (**B**) IHB-treated cells evaluated at different time intervals after drug washout at room temperature in the dark. After centrifugation, the pellet was incubated with 1 ml of 0.5% Tween-20 and 1% NGS for 15 min at room temperature. Then, the cells were incubated with a fluorescein isothiocyanate (FITC)-conjugated affinipure F(ab′)2 fragment of goat anti-mouse IgG MAb (Jackson Immuno Research Laboratories, USA) diluted 1:50 in 0.5% Tween-20 in PBS for 1 h at room temperature in the dark. The cells were finally resuspended in 2 ml of PI solution containing 2.5 μg $ml^{-1}$ PI in PBS and 25 μl of RNAase, 1 mg $ml^{-1}$ in water (Calbiochem, La Lolla, CA, USA) and stained overnight at 4°C in the dark (Erba et al, 1995).

© Cancer Research Campaign 1999 

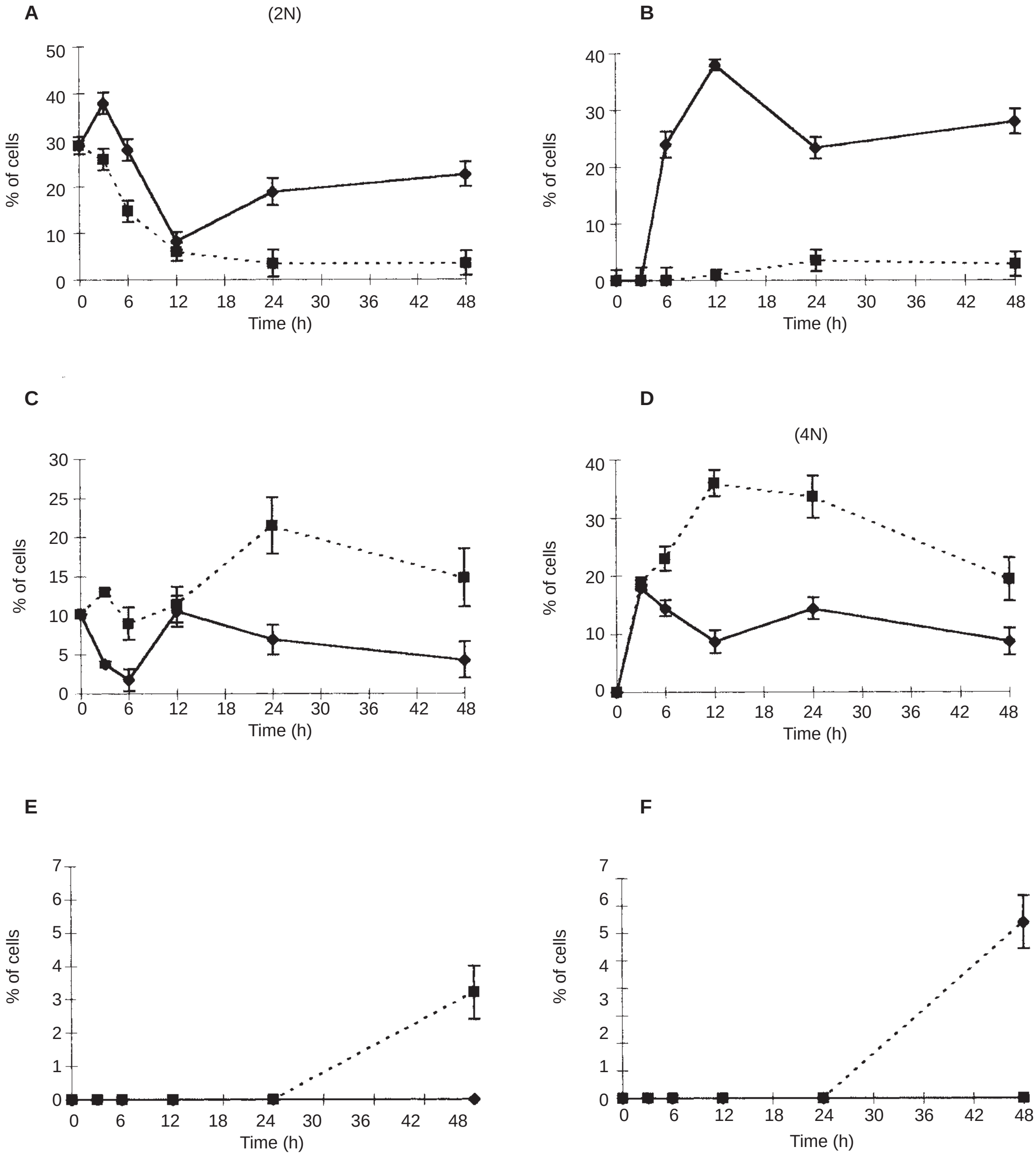

**Figure 5** Percentage of cells in relation to different DNA and BrdU content. (**A**) BrdU⁻ 2N cells (diploid $G_1$). (**B**) BrdU⁺ 2N cells (diploid $G_1$). (**C**) BrdU⁻ 4N cells (diploid $G_2M$ and $G_1$ tetraploid IHB-treated cells). (**D**) BrdU⁺ 4N cells (diploid $G_2M$ and $G_1$ tetraploid IHB-treated cells). (**E**) BrdU⁻ 8N cells ($G_2M$ cells of the new tetraploid cell population). (**F**) BrdU⁺ 8N cells ($G_2M$ cells of the new tetraploid cell population). Continuous line, control cells; dotted line, IHB-treated cells

Monoparametric DNA and biparametric BrdU/DNA analysis were performed on at least 20 000 cells for each sample by the FacSort system (Becton Dickinson). The data are the average of three replications and were analysed with Cell Quest software. The fluorescence pulses were detected using a bandpass filter, 530 ± 30 nm and 620 ± 35 nm for green and red fluorescence respectively, in combination with a 570-nm dichroic mirror. The percentage of the cell cycle phase distribution was calculated by the method of Krishan and Frei (1976).

### Cyclins A and B1/DNA staining

At the end of treatment, and at different time intervals after drug washout, the cells were fixed in 70% ethanol and stored at 4°C. The fixed cells were washed in cold PBS and permeabilized with 0.25% Triton X-100 (Sigma, St Louis, MO, USA) in PBS for 5 min in ice. Then, cells were washed in PBS and incubated with 200 μl of anti-human cyclin A, BF683 clone, or anti-human cyclin B1 monoclonal antibody, GNS-11 clone (Pharmingen, San Diego, CA, USA), at a concentration of 2.5 μg $ml^{-1}$ in PBS + 1% NGS overnight at 4°C in the dark. A blank sample was prepared by incubation of cells with 200 μl of 1% NGS in PBS or with the isotype IgG instead of the cyclin. After removing the MAb, the cells were incubated with 200 μl of FITC-conjugated affinipure F(ab′)2 fragment of goat anti-mouse IgG MAb diluted 1:50 in 0.5% Tween-20 in PBS for cyclin B1 and FITC-conjugated rat anti-mouse IgE for cyclin A for 1 h at room temperature in the dark. The cells were finally resuspended in 2 ml of PI solution

*© Cancer Research Campaign 1999*

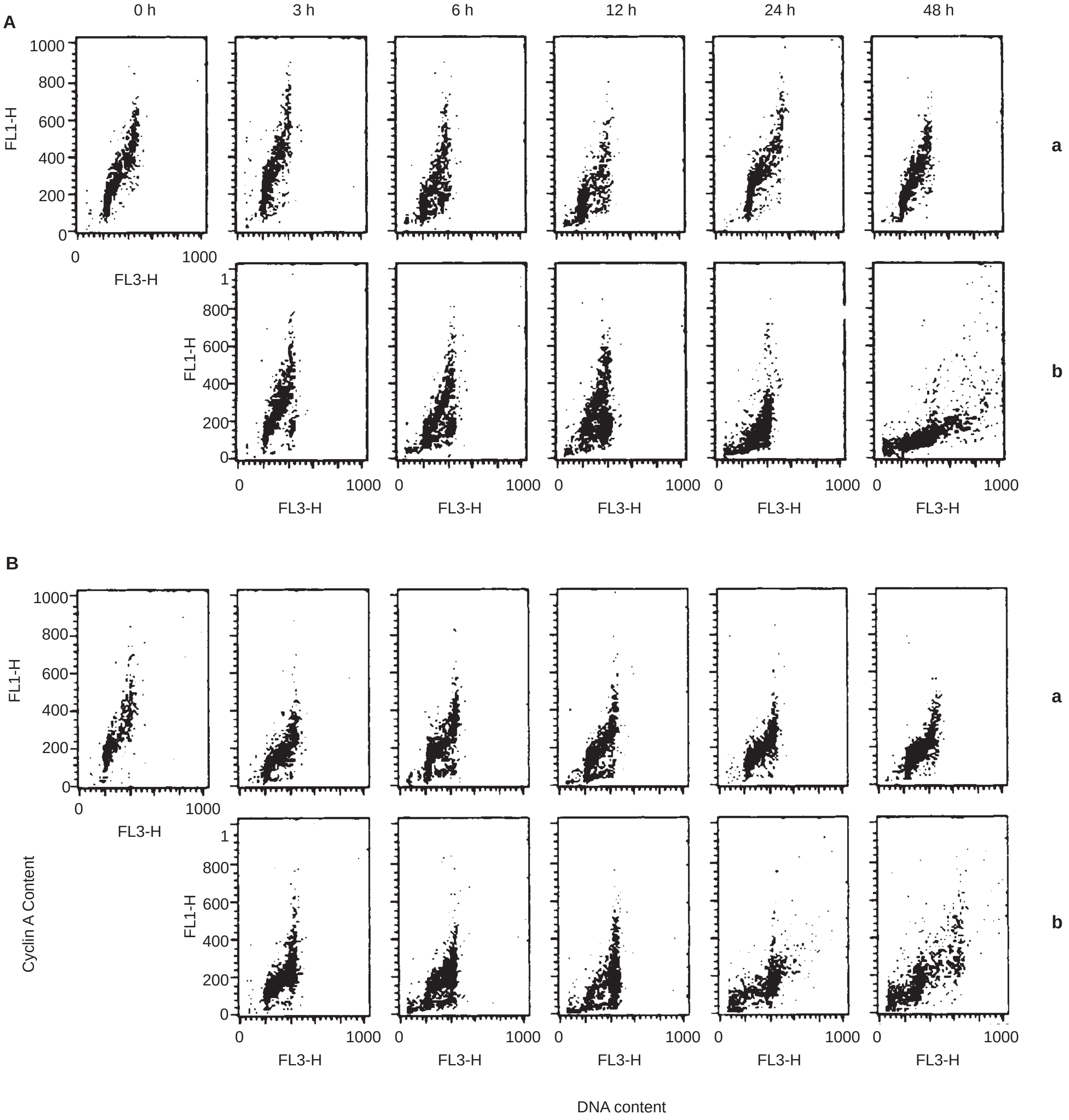

**Figure 6** Biparametric cyclin A/DNA flow cytometric analysis of (**A**) MOLT-4 and (**B**) K562 cells treated with 2 nM IHB for 1 h evaluated at different time intervals after drug-washout. a, control cells; b, IHB-treated cells containing 2.5 μg ml$^{-1}$ PI in PBS and 25 μl of RNAase, 1 mg ml$^{-1}$ in water, and stained overnight at 4°C in the dark (Gong et al, 1995; Faretta et al, 1997).

### Apoptosis/DNA staining

Cell fixation and permeabilization were performed as described for cyclin staining. Briefly, after removing Triton X-100, the cells were incubated in 50 μl of solution containing terminal-dUTP-transferase (TdT) and FITC-conjugated dUTP deoxynucleotides 1:1 in storage buffer (Boehringer Mannheim, Germany) for 60 min at 37°C in the dark. This test is based on labelling of DNA strand breaks resulting from internucleosomal cleavage by a calcium-dependent endonuclease, activated during cell death. TdT catalyses the incorporation of FITC-conjugated nucleotides to the 3′-OH free DNA ends in a template-independent manner. After washing in PBS, the cells were analysed by flow cytometry or were stained with PI solution containing 1–2 μg ml$^{-1}$ PI in PBS and 25 μl of RNAase, 1 mg ml$^{-1}$ in water, and stained overnight at 4°C in the dark (Li et al, 1995).

### Gel electrophoresis

DNA fragmentation was measured by gel electrophoresis. Briefly, $10^6$ cells were lysed in lysis buffer (5 mM Tris-HCl, pH 8; 10 mM

*© Cancer Research Campaign 1999*

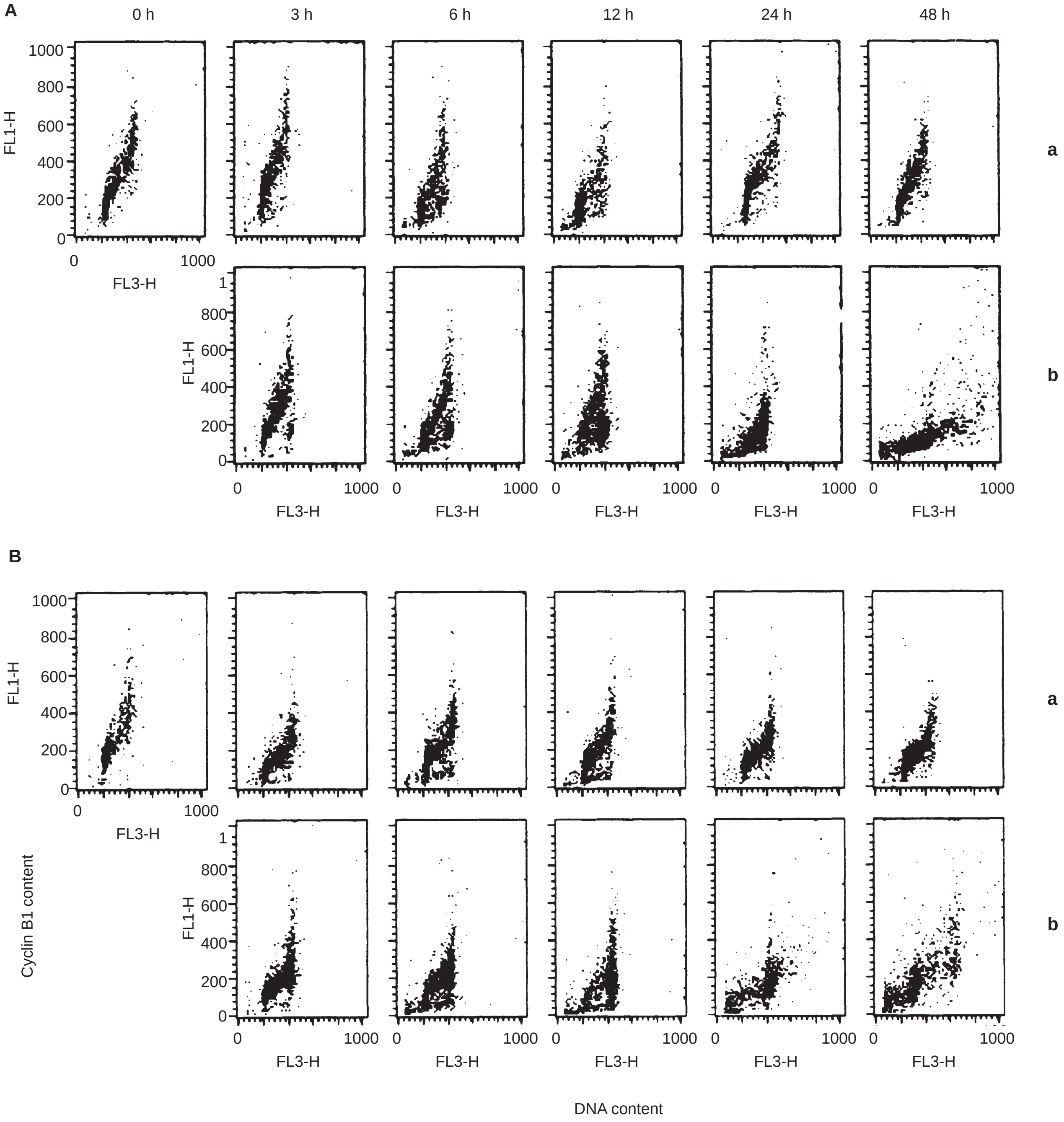

**Figure 7** Biparametric cyclin B1/DNA flow cytometric analysis of (**A**) MOLT-4 and (**B**) K562 cells treated with 2 nM IHB for 1 h evaluated at different time intervals after drug washout. a, control cells; b, IHB-treated cells

EDTA, pH 8; 0.5% Triton X-100) for 10 min on ice. The lysate was centrifuged at 12 000 ***g*** for 20 min at 4°C to remove high molecular-weight DNA. The supernatant, containing degraded DNA, RNA and cellular proteins was incubated for 1 h at 37°C with 100 mg $ml^{-1}$ RNAase and for 2 h at 50°C with 100 mg $ml^{-1}$ proteinase K. After phenol, phenol–chloroform and chloroform extraction, DNA was precipitated in ethanol, dried and resuspended in gel loading buffer. Samples were loaded on 1.5% agarose gel and DNA ladders were visualized by ethidium-bromide staining (Cohen et al, 1992).

### Immunoprecipitation

Total cell extracts were prepared from MOLT-4 and K562 after 1 h of drug treatment and at 24 h after drug washout according to standard procedures (O'Connor et al, 1993). One hundred micrograms of protein from untreated or drug-treated cells was immunoprecipitated with antibodies specific for *cdc2* (Santa Cruz Biotechnology) and collected by binding to protein A–Sepharose. After three washes in lysis buffer and two in kinase buffer, complexes were resuspended in 30 μl of kinase buffer.

 *© Cancer Research Campaign 1999*

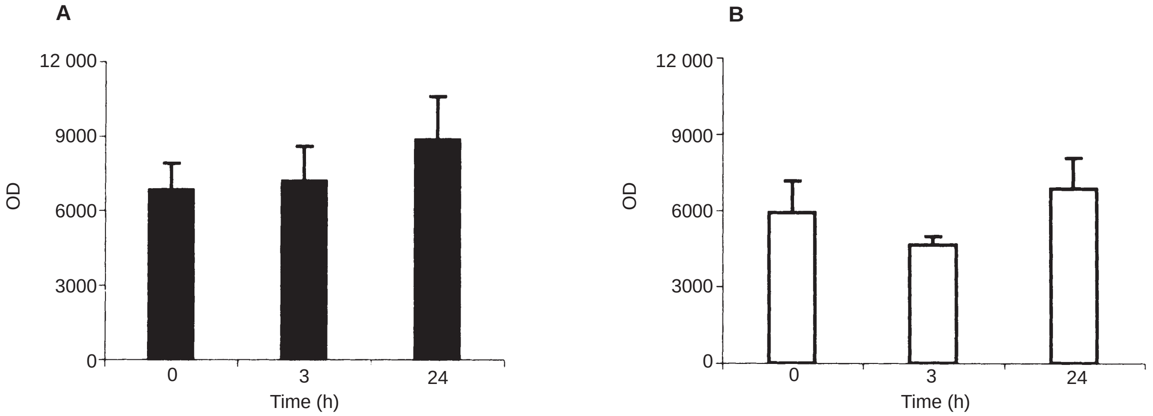

**Figure 8** Evaluation of the activity of the cyclin A and cyclin B1/Cdc2 complex quantitated by densitometric analysis of the radioactive films in (**A**) K562 and (**B**) MOLT-4 cells

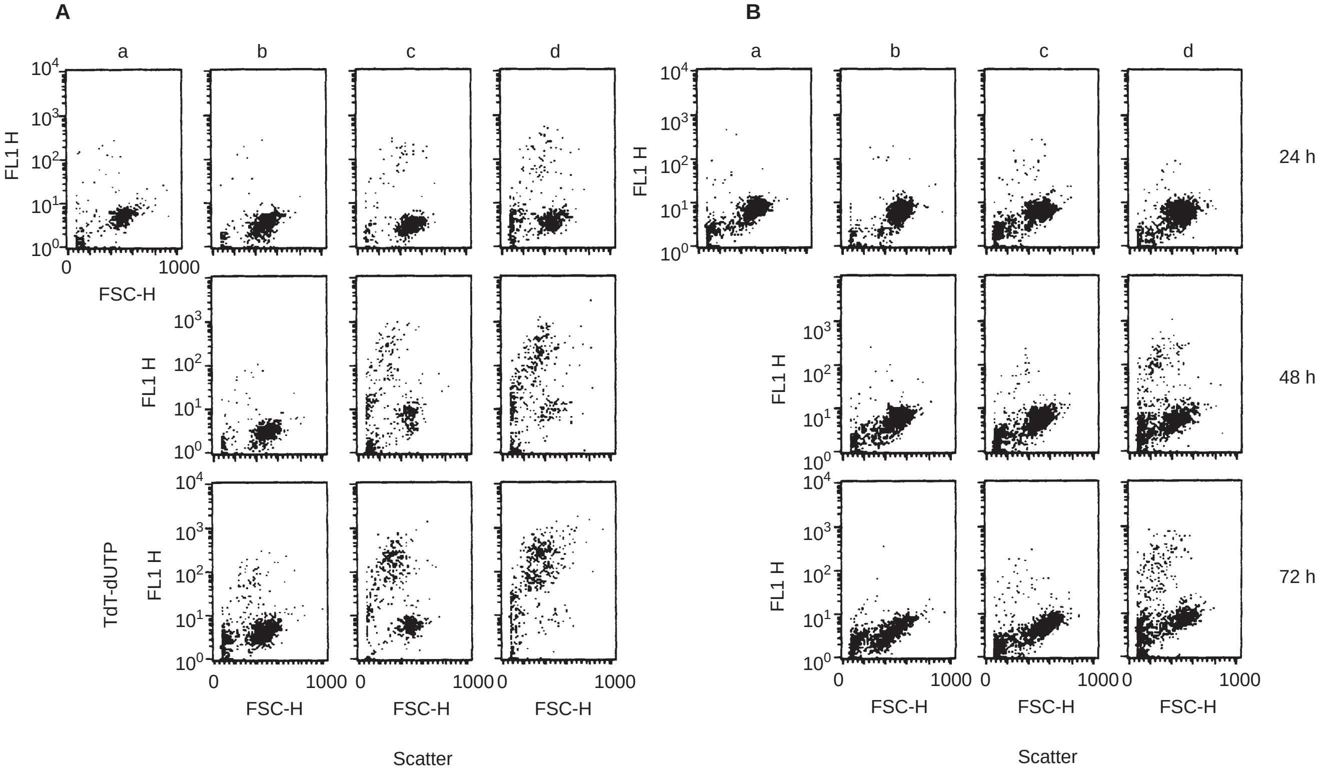

**Figure 9** Detection of apoptosis in (**A**) MOLT-4 and (**B**) K562 cells. Cells were treated for 1 h with IHB and biparametric scatter (FSC-H)/TdT-dUTP analysis was performed at different times after drug washout. a, control cells; b, 0.1 nM IHB; c, 0.5 nM IHB; d, 1 nM IHB

### In vivo kinase assay

Kinase activity was measured by incubating 100 ng of immunoprecipitated complexes containing cyclin B-*cdc2* at 30°C for 20 min in a total volume of 25 μl of kinase buffer (50 mM Tris-HCl pH 7.4, 150 mM NaCl, 0.5% Triton X-100, 10 mM magnesium chloride, 1 mM DTT) containing 2 μg of histone H1, 1 μM ATP and 5 μCi of [$\gamma$-$^{32}$P]ATP (5000 Ci mmol$^{-1}$; Amersham); 25 μl of 2 × SDS-loading buffer were added and the samples were boiled and loaded on 12% SDS-PAGE. Histone H1 was loaded as a marker of molecular weight and separately stained with Coomassie blue (Bonfanti et al, 1997).

### RESULTS

Figure 2 shows a monoparametric assessment of cell cycle distribution of LoVo, LoVo/Dx, MOLT-4 and K562 cells exposed for 1 h to IHB at a concentration that caused a similar growth inhibition. MOLT-4 and K-562 were the most sensitive, with $GI_{50}$ being approximately 1 nM; in LoVo and LoVo/Dx, $GI_{50}$ values were approximately 2 and 10 nM respectively.

IHB induced (1) an early accumulation of cells in S-phase, more evident in LoVo and MOLT-4 cells; (2) an accumulation of cells in $G_2M$, evident in all cell lines at 24 h; and (3) the appearance of cells with an increased DNA content, above that of $G_2M$ phase

© Cancer Research Campaign 1999

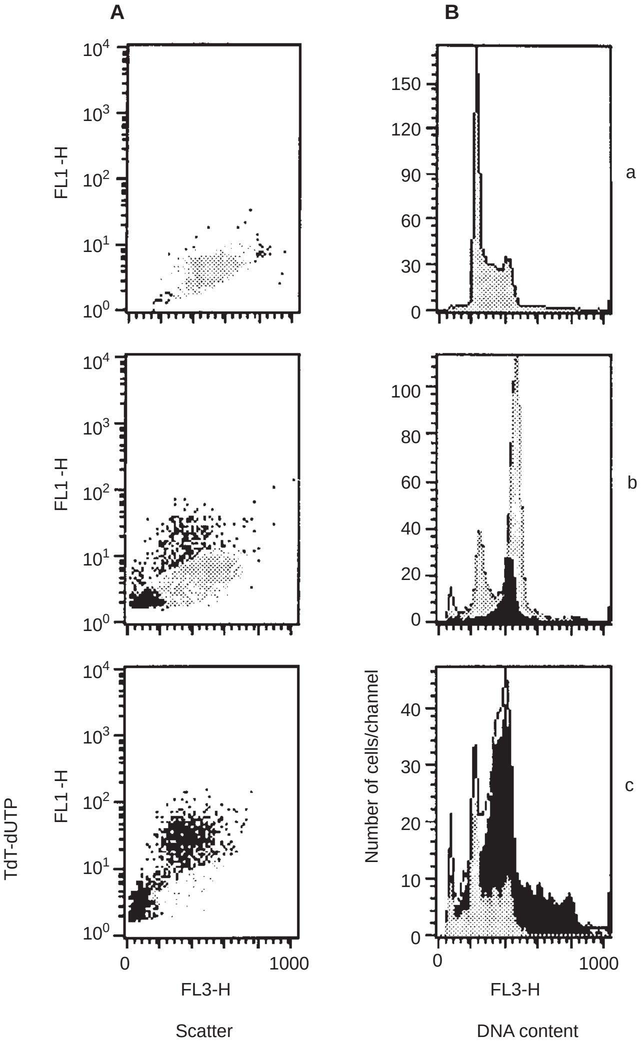

**Figure 10** Detection of apoptosis in MOLT-4 treated with 2 nM IHB for 1 h evaluated at different time intervals after drug washout. (**A**) Biparametric scatter FSC-/TdT-dUTP analysis. (**B**) DNA histograms related to viable cells or apoptotic cells. White DNA histograms, control untreated cells. (a) Grey DNA histograms, viable treated cells at 24 (b) or 72 (c) h after drug washout. Black DNA histograms, apoptotic treated cells at 24 (b) or 72 (c) h after drug washout cells, which became evident at 48 h. In order to obtain a dynamic evaluation of the drug-induced cell cycle perturbation, further experiments were conducted using a biparametric analysis of the cell cycle by means of the combined use of the BrdU/DNA staining procedure in two out of the four cell lines initially investigated, MOLT-4 and K562. As BrdU was given in the last 15 min of the 1-h incubation period with 2 nM IHB, it was possible to obtain a distinct evaluation of the cell cycle perturbations in cells that occurred in S-phase during drug treatment ($BrdU^+$ cells) and of cells that were in other phases of the cell cycle ($BrdU^-$ cells). As seen in Figures 3 and 4, 3 h after IHB washout, those cells which were in the S-phase ($BrdU^+$ cells) during drug treatment, progressed through this phase of the cell cycle more slowly than control cells. Those cells which were in the $G_2M$ phase ($BrdU^-$ cells) during drug treatment remained in $G_2M$. At 6 h after IHB washout, some $BrdU^+$ cells were still in S-phase, whereas others were blocked in the $G_2M$ phase. At the same time, in control cells, a fraction of cells that started a new cell cycle was clearly evident. At 12 h after IHB washout, all the $BrdU^+$ cells were blocked in the $G_2M$ phase of the cell cycle, whereas the $BrdU^-$ cells slowly progressed from the S phase. At 24 h, the $BrdU^+$ and the $BrdU^-$ cells were blocked in the $G_2M$ phase of the cell cycle and a new population with a tetraploid DNA content appeared. At 48 h after IHB washout, the majority of the treated cells had a tetraploid DNA content. Figure 5 shows the percentage of 2N, 4N and 8N cell populations in relation to their BrdU content. Between 24 and 48 h after IHB washout most of the treated cells were blocked in mitosis and some of them divided abnormally, giving rise to a new tetraploid cell population, more evident at 48 h after IHB washout. For this reason, in Figure 5 C and D, the percentage of 4N $BrdU^-$ and $BrdU^+$ IHB-treated cells, evaluated 24 and 48 h after drug washout, include $G_2M$ cells of the diploid population and $G_1$ cells of the new tetraploid cell population.

The biparametric cyclins/DNA analysis were then performed to characterize more precisely the cell cycle phase perturbations induced by IHB.

Figure 6 shows the levels of cyclin A in MOLT-4 and K562 cells at different time intervals after 2 nM IHB treatment. The levels of cyclin A were not changed compared with control cells, thus excluding the possibility that the slow progression of IHB treated cells through the S-phase was due to changes in the levels of this cyclin. At 12 h after drug washout, a fraction of the cyclin A-negative cell population (56% in MOLT-4, 52% in K562) was evident, indicating that IHB treated cells started to be blocked in mitosis. At 24 h after drug treatment, nearly all the cells blocked in $G_2M$ were cyclin A negative (86% in MOLT-4, 84% in K562), thus indicating that all cells were blocked in mitosis and not in the $G_2$ phase of the cell cycle.

Figure 7 shows the levels of cyclin B1 in Molt-4 and in K562 cells at different time intervals after IHB treatment. As expected, when the treated cells started to accumulate in the $G_2M$ phase of the cell cycle, the fraction of cyclin B1-positive cells increased. However, as shown for cyclin A, IHB did not cause marked changes in the levels of cyclin B1. It is interesting to note that, at 48 h after drug washout, all cells with a DNA content corresponding to $G_2M$ phase cells were cyclin B1 negative, indicating that at this time no $G_2M$ phase cells were present but only $G_1$ cells of the new tetraploid population caused by an abnormal mitosis related to IHB treatment.

Consistent with the fact that IHB blocks cells at the beginning of mitosis is the finding that both MOLT-4 or K562 cells maintained high levels of cyclin B1/*cdc2* or cyclin A/*cdc2* kinase activity as shown in Figure 8.

The data presented above indicate that the mitotic block observed within 24 h for IHB treatment was no longer present at 48 h, when the tetraploid cell population became prevalent.

Following these observations, we then investigated the mechanism of cell death induced by IHB. IHB induced apoptosis in both MOLT-4 and K562 cells, even if the level of apoptotic cells was different between the two cell lines. Figure 9 shows the results of experiments performed to detect apoptosis by using the TdT–dUTP test. At 72 h in MOLT-4 cells the amount of apoptotic cells corresponding to 15% at the IHB concentration of 0.1 nM increased to 50% and then 85% at concentrations of 0.5 and 1 nM respectively. In K562 cells at 72 h after IHB washout, only 20% of the treated cells were apoptotic.

By combining TdT–dUTP with DNA staining, it is possible to identify the cell cycle phase position of apoptotic cells. As seen

© Cancer Research Campaign 1999

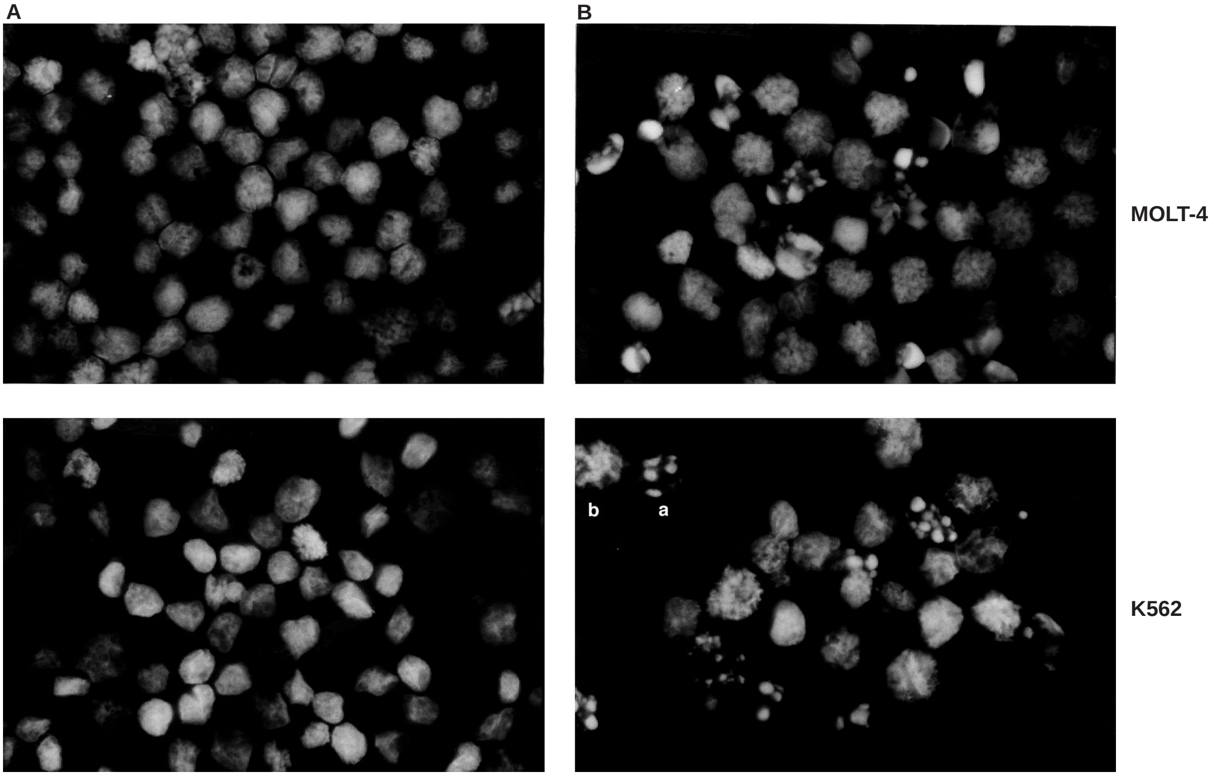

**Figure 11** UV light photomicrographs of MOLT-4 and K562 cells untreated or treated with 2 nM IHB. (**A**) Control cells; (**B**) IHB-treated cells. a, apoptotic cells with fragmented nuclei. b, cells blocked in mitosis from Figure 10, 24 h after drug washout the majority of the IHB-treated MOLT-4 cells were blocked in mitosis, as clearly shown by the grey DNA histogram. At this time the incorporation of TdT-dUTP was selective to the fraction of cells blocked in mitosis, as shown by the black DNA histogram. At 72 h after drug washout, the majority of the treated cells were positive for the TdT–dUTP test, as shown by the black DNA histogram; these cells were blocked in $G_2M$ phases or had an increased DNA content above that of the $G_2M$ phase cells. At this time, few non-apoptotic cells, with a cell cycle phase distribution similar to the control cells, were detected (grey DNA histogram). Figure 11A and B shows IHB-induced apoptosis determined by 4′,6-diamidino-2-phenyl-indole (DAPI) and sulphorhodamine 101 staining. IHB induced morphological changes associated with apoptosis, nuclear condensation and formation of apoptotic bodies, and cells blocked in prophase were clearly evident (Figure 11B, upper panel). At later time points, the number of apoptotic cells increased (Figure 11, lower panel). Further demonstration of apoptosis was obtained by looking at DNA laddering, as shown in Figure 12. In MOLT-4 cells, DNA fragmentation was evident at 0.5 and 1 nM IHB at both 24 and 48 h after IHB washout, whereas in the K562 cells DNA fragmentation was not evident until 48 h after drug washout and only at a concentration of 1 nM.

### DISCUSSION

The present study reports the perturbations of the cell cycle and the mechanism of cell death induced by IHB, a novel marine-derived anti-tumour compound. The observed effects, which can be summarized as a delayed progression through S-phase, a $G_2M$ block and the appearance of a tetraploid population, may well be related to the previously reported data indicating that IHB targets the tubulin network (García-Rocha et al, 1996). García Rocha et al (1996) reported that 2 μM IHB inhibited in vitro tubulin polymerization. The same authors reported that 7 μM IHB for 2 h significantly disrupted the microtubule cytoskeleton of COS-1 cell line as assessed by morphological examination after tubulin staining. It is possible that both S-phase delayed progression and the $G_2M$ block observed in the present study are related to these biochemical effects reported by García-Rocha et al (1996). However, it should be noted that the IHB concentrations used in this study were at least 1000 times lower than those used in the mentioned studies. The discrepancy between high cytotoxic potency ($IC_{50}$ in nM range) and weak inhibition of in vitro tubulin polymerization ($K_i$ in μM range) has already been described for the structurally similar compound, homohalichondrin B (Bai et al, 1991). These authors also characterized the ability of this compound to inhibit tubulin-dependent GTP hydrolysis, but have not provided any explanation for the relatively high concentrations required for the in vitro effects. It might be that the inhibition of tubulin polymerization is dependent upon other factors present in intact cells, but not in the in vitro assays. Alternatively, other mechanisms of cytotoxicity should be considered.

There is little knowledge on the binding of IHB to tubulin, whereas much more information is available for homohalichondrin B, which is structurally similar to IHB. Luduena et al (1993) found that homohalichondrin B has a distinct mechanism of interaction with tubulin molecules compared with vinca alkaloids, maytansine, dolastatin 10 or phomopsin A. In particular, homohalichondrin B differed from other anti-mitotic drugs in that it enhanced

*© Cancer Research Campaign 1999* 

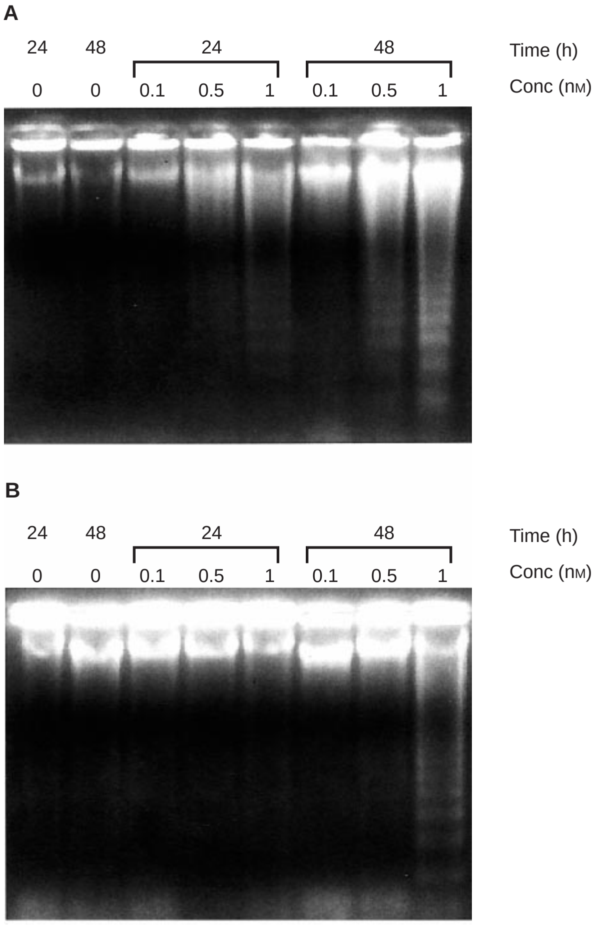

**Figure 12** Gel electrophoresis of DNA extracted from (**A**) MOLT-4 and (**B**) K562 cells treated with different concentrations of IHB at different times after drug washout exposure of hydrophobic areas on tubulin. It remains to be established if these differences also apply to IHB and if they are relevant in determining differences in the pharmacological properties between this compound and the other inhibitors of tubulin (Jordan et al, 1991).

A particularly striking finding of the present study is that IHB induces both the appearance of a tetraploid cell population and apoptosis. The mechanism of induction of polyploidy by drugs is generally poorly understood (Watters et al, 1994; Cross et al, 1995). It has recently been reported that the expression of mutated p53 or the loss of wild-type (wt) p53 induces the appearance of polyploid cells following γ-irradiation (Peled et al, 1996). In the present study, we found that IHB was able to induce polyploidy in both wt p53-expressing cells, such as LoVo and MOLT-4 cells, and in cells not expressing p53, such as K562 cells. Therefore, the induction of polyploidy by IHB does not appear to be mediated by p53 expression.

Nor can we draw any conclusion regarding the role of p53 in IHB-induced apoptosis. In fact, we found that IHB induced apoptosis to a much greater extent in MOLT-4 cells, which express wt p53, than in K562 cells and markedly increased the expression of this protein after IHB exposure (data not shown). It should also be noted, however, that in the K562 leukaemic cell line a fraction of apoptotic cells was observed after IHB treatment, but not with other anti-cancer drugs, e.g. *cis*-DDP (data not shown). It appears, therefore, that IHB-induced apoptosis is not necessarily or totally p53 dependent, as is the case for other anti-cancer agents (Strasser et al, 1994; De Feudis et al, 1996).

In conclusion, the present study reports the first characterization of cell cycle perturbation and cell death mechanism by the novel marine natural product IHB. This compound has been recently seen to be active in vivo against rodent tumours and human xenografts and is a potential candidate drug for clinical studies. In the past, the clinical development of halichondrin B has been hampered by the very low availability of this natural product. The greater availability of IHB makes this compound more accessible for preclinical development and to identify appropriate tumour targets for the clinic.

### ACKNOWLEDGEMENT

The generous contribution of the Italian Association for Cancer Research, Milan, Italy is gratefully acknowledged.

### REFERENCES

Bai R, Paull KD, Herald CL, Malspeis L, Pettit GR and Hamel E (1991) Halichondrin B and Homohalichondrin B, marine natural products binding in the vinca domain of tubulin. *J Biol Chem* **266**: 1582–1589

Bonfanti M, Taverna S, Salmona M, D'Incalci M and Broggini M (1997) $p21^{waf1}$-derived peptides linked to an internalization peptide inhibit human cancer cell growth. *Cancer Res* **57**: 1442–1446

Cohen GM, Sun XM, Snowden RT, Dinsdale D and Skilleter DN (1992) Key morphological features of apoptosis may occur in the absence of internucleosomal DNA fragmentation. *Biochem J* **286**: 331–334

Cross SM, Sanchez CA, Morgan CA, Schimke MK, Ramel S, Idzerda RL, Raskind WH and Reid BJ (1995) A p53-dependent mouse spindle checkpoint. *Science* **267**: 1353–1356

De Feudis P, D'Incalci M and Broggini M (1996) Block of bcr-abl expression and induction of apoptosis by cisplatinum in a human chronic myeloid leukemia cell line. *Apoptosis* **1**: 159–164

Erba E, Mascellani E, Pifferi and D'Incalci M (1995) Comparison of cell-cycle perturbations induced by the DNA-minor groove alkylator tallimustine and by melphalan in the SW626 cell line. *Int J Cancer* **62**: 170–175

Faretta M, Bergamaschi D, Taverna S, Ronzoni S, Pantarotto M, Mascellani E, Cappella P, Ubezio P and Erba E (1998) Characterization of cyclin B1 expression in human cancer cell lines by a new three-parameter BrdUrd/cyclin/DNA analysis. *Cytometry* **31**: 53–59

García-Rocha M, García-Gravalos MD and Avila J (1996) Characterisation of antimitotic products from marine organism that disorganise the microtubule network: ecteinascidin 743, isohomohalichondrin-B and LL-15. *Br J Cancer* **73**: 875–883

Gong J, Traganos F and Darzynkiewicz Z (1995) Discrimination of G2 and mitotic cells by flow cytometry based on different expression of cyclins A and B1. *Exp Cell Res* **220**: 226–231

Gorczyca W, Bruno S, Darzynkiewicz RJ, Gong J and Darzynkiewicz Z (1992) DNA strand breaks occurring during apoptosis: their early in situ detection by the terminal deoxynucleotidyl transferase and nick translation assays and prevention by serine protease inhibitors. *Int J Oncol* **1**: 639–648

Jordan MA, Thrower D and Wilson L (1991) Mechanism of inhibition of cell proliferation by *vinca* alkaloids. *Cancer Res* **51**: 2212–2222

Litaudon M, Hart JB, Blunt JW, Lake RJ and Munro MHG (1994) Isohomohalichondrin B, a new antitumour polyether macrolide from the New Zealand deep-water sponge *Lissodendoryx* sp. *Tetra Lett* **35**: 9435–9438

Lowe SW, Ruley HE, Jacks T and Hosman DE (1993) p53-dependent apoptosis modulates the cytotoxicity of anticancer agents. *Cell* **74**: 957–967

Ludueña RF, Roach MC, Prasad V and Pettit GR (1993) Interaction of halichondrin B and homohalichondrin B with bovine brain tubulin. *Pract Pharmacol* **45**: 421–427

Li X, Traganos F, Melamed MR and Darzynkiewicz Z (1995) Single-step procedure for labeling DNA strand breaks with fluorescein- or Bodipy-conjugated

© Cancer Research Campaign 1999

deoxynucleotides: detection of apoptosis and bromodeoxyuridine incorporation. *Cytometry* **20**: 172–180

O'Connor PM, Ferris DK, Pagano M, Draetta G, Pines J, Hunter T, Longo DL and Kohn KW (1993) G2 delay induced by nitrogen mustard in human cells affects cyclin A/Cdk2 and Cyclin B1/*cdc2*-kinase complexes differently. *J Biol Chem* **268**: 8298–8308

Peled A, Schwartz D, Elkind NB, Wolkowicz R, Li R and Rotter V (1996) The role of p53 in the induction of myelomonocytic leukemic M1/2 cells. *Oncogene* **13**: 1677–1685

Strasser A, Harris AW, Jacks T and Cory S (1994) DNA damage can induce apoptosis in proliferating lymphoid cells via p53-independent mechanism inhibitable by Bcl-2 (see comments). *Cell* **79**: 329–339

Watters DJ, Beamish HJ, Marshall KA, Gardiner RA, Seymour GJ and Lavin MF (1994) Accumulation of HL-60 leukemia cells in G2/M and inhibition of cytokinesis caused by two marine compounds, bistratene A and cycloxazoline. *Cancer Chemother Pharmacol* **33**: 399–409

*© Cancer Research Campaign 1999*

## References

[PDF_00941] Bonfanti M., Taverna S., Salmona M., D'Incalci M., Broggini M. (1997). p21WAF1-derived peptides linked to an internalization peptide inhibit human cancer cell growth.. Cancer Res.

[PDF_00944] Cohen G. M., Sun X. M., Snowden R. T., Dinsdale D., Skilleter D. N. (1992). Key morphological features of apoptosis may occur in the absence of internucleosomal DNA fragmentation.. Biochem J.

[PDF_00947] Cross S. M., Sanchez C. A., Morgan C. A., Schimke M. K., Ramel S., Idzerda R. L., Raskind W. H., Reid B. J. (1995). A p53-dependent mouse spindle checkpoint.. Science.

[PDF_00953] Erba E., Mascellani E., Pifferi A., D'Incalci M. (1995). Comparison of cell-cycle phase perturbations induced by the DNA-minor-groove alkylator tallimustine and by melphalan in the SW626 cell line.. Int J Cancer.

[PDF_00956] Faretta M., Bergamaschi D., Taverna S., Ronzoni S., Pantarotto M., Mascellani E., Cappella P., Ubezio P., Erba E. (1998). Characterization of cyclin B1 expression in human cancer cell lines by a new three-parameter BrdUrd/cyclin B1/DNA analysis.. Cytometry.

[PDF_00960] García-Rocha M., García-Gravalos M. D., Avila J. (1996). Characterisation of antimitotic products from marine organisms that disorganise the microtubule network: ecteinascidin 743, isohomohalichondrin-B and LL-15.. Br J Cancer.

[PDF_00964] Gong J., Traganos F., Darzynkiewicz Z. (1995). Discrimination of G2 and mitotic cells by flow cytometry based on different expression of cyclins A and B1.. Exp Cell Res.

[PDF_00971] Jordan M. A., Thrower D., Wilson L. (1991). Mechanism of inhibition of cell proliferation by Vinca alkaloids.. Cancer Res.

[PDF_00976] Lowe S. W., Ruley H. E., Jacks T., Housman D. E. (1993). p53-dependent apoptosis modulates the cytotoxicity of anticancer agents.. Cell.

[PDF_00978] Ludueña R. F., Roach M. C., Prasad V., Pettit G. R. (1993). Interaction of halichondrin B and homohalichondrin B with bovine brain tubulin.. Biochem Pharmacol.

[PDF_01006] O'Connor P. M., Ferris D. K., Pagano M., Draetta G., Pines J., Hunter T., Longo D. L., Kohn K. W. (1993). G2 delay induced by nitrogen mustard in human cells affects cyclin A/cdk2 and cyclin B1/cdc2-kinase complexes differently.. J Biol Chem.

[PDF_01010] Peled A., Schwartz D., Elkind N. B., Wolkowicz R., Li R., Rotter V. (1996). The role of p53 in the induction of polyploidity of myelomonocytic leukemic M1/2 cells.. Oncogene.

[PDF_01013] Strasser A., Harris A. W., Jacks T., Cory S. (1994). DNA damage can induce apoptosis in proliferating lymphoid cells via p53-independent mechanisms inhibitable by Bcl-2.. Cell.

[PDF_01016] Watters D. J., Beamish H. J., Marshall K. A., Gardiner R. A., Seymour G. J., Lavin M. F. (1994). Accumulation of HL-60 leukemia cells in G2/M and inhibition of cytokinesis caused by two marine compounds, bistratene A and cycloxazoline.. Cancer Chemother Pharmacol.

